# Gastric lactobezoar - a rare disorder?

**DOI:** 10.1186/1750-1172-7-3

**Published:** 2012-01-04

**Authors:** Peter Heinz-Erian, Ingmar Gassner, Andreas Klein-Franke, Veronika Jud, Rudolf Trawoeger, Christian Niederwanger, Thomas Mueller, Bernhard Meister, Sabine Scholl-Buergi

**Affiliations:** 1 Department of Pediatrics, Medical University, Anichstrasse 35, A-6020 Innsbruck, Austria

**Keywords:** gastric lactobezoar, formula preparation, acute abdomen, dehydration, immature gastrointestinal functions, underdiagnosed entity, conservative management

## Abstract

Gastric lactobezoar, a pathological conglomeration of milk and mucus in the stomach of milk-fed infants often causing gastric outlet obstruction, is a rarely reported disorder (96 cases since its first description in 1959). While most patients were described 1975-1985 only 26 children have been published since 1986. Clinically, gastric lactobezoars frequently manifest as acute abdomen with abdominal distension (61.0% of 96 patients), vomiting (54.2%), diarrhea (21.9%), and/or a palpable abdominal mass (19.8%). Respiratory (23.0%) and cardiocirculatory (16.7%) symptoms are not uncommon. The pathogenesis of lactobezoar formation is multifactorial: exogenous influences such as high casein content (54.2%), medium chain triglycerides (54.2%) or enhanced caloric density (65.6%) of infant milk as well as endogenous factors including immature gastrointestinal functions (66.0%), dehydration (27.5%) and many other mechanisms have been suggested. Diagnosis is easy if the potential presence of a gastric lactobezoar is thought of, and is based on a history of inappropriate milk feeding, signs of acute abdomen and characteristic features of diagnostic imaging. Previously, plain and/or air-, clear fluid- or opaque contrast medium radiography techniques were used to demonstrate a mass free-floating in the lumen of the stomach. This feature differentiates a gastric lactobezoar from intussusception or an abdominal neoplasm. Currently, abdominal ultrasound, showing highly echogenic intrabezoaric air trapping, is the diagnostic method of choice. However, identifying a gastric lactobezoar requires an investigator experienced in gastrointestinal problems of infancy as can be appreciated from the results of our review which show that in not even a single patient gastric lactobezoar was initially considered as a possible differential diagnosis. Furthermore, in over 30% of plain radiographs reported, diagnosis was initially missed although a lactobezoar was clearly demonstrable on repeat evaluation of the same X-ray films. Enhanced diagnostic sensitivity would be most rewarding since management consisting of cessation of oral feedings combined with administration of intravenous fluids and gastric lavage is easy and resolves over 85% of gastric lactobezoars. In conclusion, gastric lactobezoar is a disorder of unknown prevalence and is nowadays very rarely published, possibly because of inadequate diagnostic sensitivity and/or not yet identified but beneficial modifications of patient management.

## Introduction

Gastric lactobezoar (GLB) is a type of acid-insoluble bezoar characterized by its composition of milk and mucus components [1] and localization in the stomach as a free-floating lump. Lactobezoars also differ from other bezoar types such as tricho-, phyto-, mixed food- or medication-bezoars [2] by their almost invariable occurrence during early age, indeed the majority of lactobezoars reported in the literature has been diagnosed in premature neonates [2-5]. While most lactobezoars are located in the stomach, some of them have also been found in the upper [6-8] and lower intestine [9], all of them being manifestations of the inspissated milk syndrome [10]. No genetic associations have been reported so far.

This paper summarizes data of 96 patients with GLB from 40 publications identified by searches in PubMed, EMBASE, DIALINDEX, Biosis Previews, CAB Abstracts, Pascal, Sci Search, American Academy of Pediatrics Search Site, JSTOR, LactMed, Lange Case Files, Up To Date, Ovid and Cochrane data bases. Our major purpose is to 1) review the current knowledge about this disorder, 2) alert physicians to the possibility that GLB may be a differential diagnosis of acute abdomen, 3) discuss why GLBs are now so rarely reported, and 4) emphasize early conservative treatment instead of primary endoscopic or surgical interventions.

## Epidemiology

The true prevalence of GLB is unknown. Most of the 96 cases reported were diagnosed in North American Hospitals (USA 76 patients, Canada 1). Additional patients were published by authors from South Africa (4), France (4), UK (4), Austria (3), Germany (1), Israel (1), The Netherlands (1) and Saudi Arabia (1). Of the 51 cases, in whom gender was reported, 22 were females, 29 males. 61 of the 87 patients (70.1%), in whom the age at manifestation of the GLB was documented, were 30 days old or younger. In 20 further patients a GLB became manifest between 31 and 365 days and in 5 toddlers between the age of 440 and 1080 days. We found only 1 publication on a GLB-patient beyond toddlers age [11]. Gestational age was documented in 79.2% (76/96) of the published children, of whom 76.7% were born prematurely whereas 23.3% were term. The mean gestational age of these 76 babies was 33 ± 4 weeks (range 24-41) whereby no difference between females and males was found. Publications where both the newborn's gestational age and birth weight were documented (74/96 patients; 77.1%) show that 70.7% of these children had a birth weight appropriate for gestational age whereas 29.3% were small for gestational age.

## Clinical presentation

Clinical symptoms & signs of GLB are summarized in Table 1. GLBs were found in asymptomatic patients [3,4] or became manifest by predominantly gastrointestinal (GI) symptomatology which affected 77/96, i.e. over 80% of all patients (Table 1). Surprisingly, not a single patient was reported to have apparent hematemesis although signs of mechanical irritation and ulceration of the mucosa by GLB and in 7 cases even gastric perforation [1,3,4,12] were described. GI symptoms were accompanied by disturbances of fluid and metabolic balance in 37/96 children (38.5%). Respiratory symptoms including apnea were a rather prominent manifestation and GLB was found to be the only related etiology in at least 20 reported patients (22.0%), all of them premature infants [3,4,13,14]. Amongst cardiocirculatory problems, bradycardia was encountered predominantly in premature babies while tachycardia prevailed in mature infants and toddlers, and was in the latter group often associated with anemia [15,16]. Neurological [11,17,18] and infectious/allergic manifestations [4,19] were also described with GLB although the mechanisms of these associations are currently not understood.

**Table 1:** Clinical symptoms & signs of gastric lactobezoar

Symptoms & signs	patients reported^*)^	presenting symptom & sign^*)^
No symptoms & signs	5 (5.2)	-
**Gastrointestinal**		
Abdominal distension	59 (61.0)	45 (46.9)
Vomiting/regurgitation	52 (54.2)	42 (43.8)
Gastric residuals	26 (27.1)	23 (24.0)
Milk curds	9 (9.4)	2 (2.1)
Palpable abdominal mass	19 (19.8)	4 (4.2)
Weight loss/failure to thrive	9 (9.4)	1 (1.0)
Diarrhea	21 (21.9)	18 (18.9)
Melena/positive hematest	7 (7.3)	4 (4.2)
Constipation	2 (2.1)	-
**Fluid/Metabolic**		
Dehydration	24 (25.0)	8 (8.3)
Increased sweating	1 (1.0)	-
Edema	3 (3.1)	1 (1.0)
Metabolic acidosis	1 (1.0)	-
**Cardiocirculatory/Respiratory**		
Respiratory distress	16 (16.7)	7 (7.3)
Apnea	6 (6.3)	4 (4.2)
Cardiocirculatory symptoms	16 (16.7)	6 (6.3)
Anemia	6 (6.3)	4 (4.2)
**Infectious/Allergic**		
Infection	6 (6.3)	-
Fever	6 (6.3)	3 (3.1)
Ekzema	1 (1.0)	-
Allergic symptoms&signs	4 (4.2)	-
**Neurological**		
Lethargy	7 (7.3)	3 (3.1)
Crying	2 (2.1)	1
Irritability	6 (6.3)	4 (4.2)
Temper tantrums	1 (1.0)	-
Cerebral seizures	1 (1.0)	1 (1.0)

*) # show frequencies of parameters indicated in 96 patients from 40 literature reports, numbers in parentheses show percentages per 96 patients

## Pathogenesis

GLBs are thought to result from the coagulation of milk and mucous proteins following disturbed gastric function. Observed or postulated conditions favouring GLB formation included both exogenous and endogenous factors as summarized in Table 2.

**Table 2:** Pathogenetic factors reported/postulated with gastric lactobezoar

Pathogenetic factor	reported^*)^	postulated^$)^
**Exogenous**		
"Overconcentrated" formula	18 (18.8)	14 (35.0)
Calorie content > 80 kcal/100 ml	63 (65.6)	6 (15.0)
Special LBW formula	52 (54.2)	10 (25.0)
Breast milk fortifier	4 (4.2)	5 (12.5)
Protein concentration > DRI^#)^	9 (9.4)	4 (10.0)
Casein predominance > 60%	52 (54.2)	8 (20.0)
Cow's milk protein	1 (1.0)	3 (7.5)
Medium chain triglycerides	52 (54.2)	11 (27.5)
Increased Ca-content of formula	-	3 (7.5)
Increased Ca/P ratio of formula	-	3 (7.5)
Silica (Gelopectose)	3 (3.1)	3 (7.5)
Alginate (Gaviscon)	2 (2.1)	1 (2.5)
Pectin-lignin-cellulose(Aroban)	1 (1.0)	2 (5.0)
Atropin (e.g. Eumydrin^®^)	2 (2.1)	3 (7.5)
Antacids (Sucralfate^® ^Aluminium hydroxide etc.)	3 (3.1)	5 (12.5)
Modes of feeding - oral - gavage - bolus - continuous	29 (30.2)	7 (17.5)
Age < 14 days at start of > 80 kcal formula	35 (36.5)	6 (15.0)
Supine body position (poor gastric mixing)	9 (9.4)	3 (7.5)
Phototherapy prior to lactobezoar diagnosis	16 (16.7)	3 (7.5)
Increased sweating (due to hot weather)	1 (1.0)	2 (5.0)
**Endogenous**		
Prematurity	54 (56.3)	21 (52.5)
Low birth weight (< 2500 gm)	52 (54.2)	8 (20.0)
Dehydration	12 (12.5)	11 (27.5)
Increased gastric absorption of fluids	-	3 (7.5)
Decreased global gastric secretion	3 (3.1)	2 (5.0)
Interaction of formula with gastric secretion	-	2 (5.0)
Increased osmolality of gastric content	1 (1.0)	1 (2.5)
Delayed gastric emptying	3 (3.1)	16 (40.0)
Postprandial time course of gastric acidity	-	1 (2.5)
Decreased "digestive capacity"	-	3 (7.5)
Decreased enzyme activity	-	2 (5.0)
Gastric Ca secretion	-	3 (7.5)
Gastric Ca+fat/protein precipitates	1 (1.0)	3 (7.5)
Respiratory distress syndrome	14 (14.6)	6 (15.0)
Birth asphyxia	9 (9.4)	4 (10.0)
Systemic infection	10 (10.4)	4 (10.0)

* ) indicates pathogenetic factors documented in #/96 patients (numbers in parentheses are percentages per 96 patients)

^$ ^) indicates postulated factors in 40 literature reports (numbers in parentheses are percentages per 40 reports)

^#^) DRI dietary reference intake (age-adapted)

**Figure 1:**
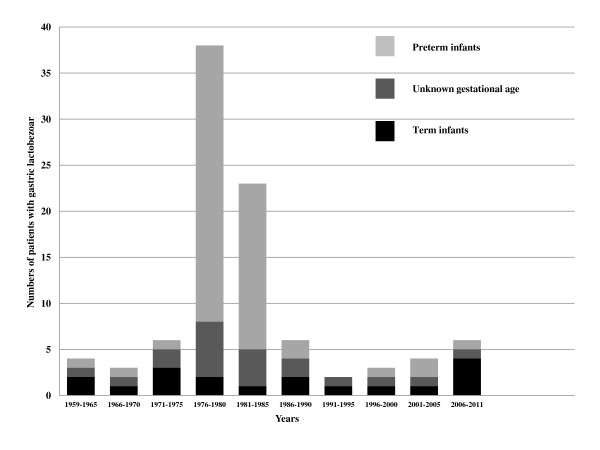
**Cases of GLB reported in the literature between 1959 and 2010**.

Exogenous influences were related to 1) composition of formula, 2) medications inhibiting gastric secretion and motility, 3) modalities of feeding, and 4) conditions causing dehydration. Data on the composition of GLB are scarce. Levkoff [1] described a gelatinous, amorphous material which microscopically contained "protein, few cells, moderate numbers of bacteria, and crystals of cholesterol, lactose and triple phosphate". Later Erenberg [5] found elevated long chain triglyceride (LCT) and decreased medium chain triglyceride (MCT) concentrations in GLBs indicating a better absorption of MCTs by the stomach. Absorbed MCTs delay gastric emptying [19,20] and thus facilitate GLB formation. Therefore the introduction of MCT-enriched formula in the late 1970ies may have been one factor causing a striking temporary increase of GLB cases at that time (Figure 1). Why, after 1985, numbers of reported GLB-patients have - despite continued MCT-rich nutrition - returned to levels before 1975 will, however, remain difficult to clarify.

With over-concentrated formula resulting from erroneous preparation predominantly during the 1960ies and 1970ies (Table 2) the content of calories and nutrients clearly exceeded age-related dietary reference intakes (DRI) [21,22]. This may be particularly true for protein as can be calculated from the data given in the literature for at least 9 cases [4,23-26]. Also, the introduction of special LBW formulas may have to do with protein concentrations too high for the digestive capacity of premature infants [27-29]. The quality of milk protein [10,13] was believed to be another factor in the formation of GLB: until 1980 commercial milk formulas contained protein with 80% caseine and 20% whey, contrary to human breast milk where this ratio is approximately 40:60. Because until then lactobezoars had only been reported in infants fed with these "non-humanized" formulas and not in those with human breast milk, it was thought useful to adapt the caseine: whey ratio to that in human milk. While these whey-protein-predominant formulas were initially reported to prevent GLB [13] the subsequent years have shown that GLBs also form with humanized formulas [30] and even with human breast milk [31].

Besides nutritional factors, medications used to reduce vomiting and diarrhoea [2,32-34] or to antagonize gastric secretion and motility (Table 2) have been associated with GLB formation [2,15,23,32]. These agents cause increased coagulation of gastric protein leading - together with elevated concentrations of calcium, phosphorus and fat - to curds with enhanced tension as the basic constituents of GLB [3,10,22].

Modalities of feeding are also discussed: firstly, the postnatal age at which enteral feeds were started and the speed at which food volumes and concentrations were advanced were suspected to influence GLB formation [3,4,35]. Furthermore, the question whether enteral continuous drip or intermittent bolus feeding should be preferred remains undetermined [36] although in this review we found 34 patients with GLB after enteral continuous drip but only 22 babies after bolus feeding (Table 2), suggesting a lower risk in the latter. With regard to technique, supine body posture during feeding was associated with air-accumulation in the prepyloric antrum impeding the passage of gastric content towards the duodenum [37]. However, despite this seemingly logical explanation, the literature mentioned only 9 cases of GLB in babies fed in the supine position. A fourth group of exogenous factors responsible for GLB-formation were conditions leading to dehydration including - besides inadequate fluid intake - phototherapy [30,38] and hot weather [15] (Table 2).

The predominant endogenous risk factors for GLB formation, prematurity and low birth weight [3,10] imply many poorly developed essential physiological functions (Table 2) comprising e.g. low "digestive capacity" due to reduced gastric acid production and pepsin activity [39-41]. These functional insufficiencies may be drastically enhanced by a very labile state of tissue hydration readily permitting the initiation of a vicious cycle [42]: milk formulae which put too much load upon the capacities of the GI-tract cause vomiting and/or diarrhea. This produces diminution of extracellular fluid volume (dehydration). To accommodate for dehydration, water is excessively absorbed from the GI-tract which leads to GLB-formation and often gastric outlet obstruction followed again by vomiting and dehydration.

## Diagnosis

The essential criteria for diagnosing GLB are summarized in Table 3. A precise nutritional history was found to be crucial in revealing mistakes of formula preparation and feeding modalities [3,4]. Often, reported decreased intakes or increased losses of fluid explained GLB-related dehydration which was in more serious cases even associated with cardiocirculatory problems [4,23,43]. Signs of respiratory distress were sometimes a manifestation of mechanical disturbance of lung function by the bezoar [2,15-17] and were in nearly 20% of cases associated with a palpable abdominal mass.

**Table 3:** Diagnostic parameters obtained in the investigation of gastric lactobezoar

Diagnostic criteria	Diagnostic information	patients reported^*)^
**Patient history**	- calorie content > 80 kcal/100 ml formula	69 (71.9)
	- mode/technique of feeding	63 (65.6)
	- food additives (milk fortifiers, anti-reflux preparations)	7 (7.3)
	- decreased fluid intake/24 hrs	17 (17.7)
	- increased fluid loss (vomiting, diarrhea, sweating)	71 (74.0)
	- medications antagonizing gastric secretion & motility	7 (7.3)
**Physical examination**	- signs of acute abdomen	67 (69.8)
	- palpable abdominal mass	19 (19.8)
	- physical signs of dehydration	16 (16.7)
	- signs of cardiocirculatory distress	17 (17.7)
	- signs of respiratory distress	15 (15.6)
**Laboratory**	- parameters of blood loss (↓ hemoglobin, ferritinetc.)	6 (6.3)
	- hematest positive stool	5 (5.2)
	- parameters of dehydration (↑ hematocrit, BUN etc.)	12 (12.5)
**Diagnostic**	- abdominal ultrasound imaging	12 (12.5)
	- plain abdominal radiography	62 (64.6)
	- air contrast radiography	18 (18.8)
	- clear fluid feed radiography	14 (14.6)
	- opaque contrast medium radiography	10 (10.4)
	- X-ray video imaging	13 (13.5)
**Gastroscopy**		2 (2.1)
**Surgery**		9 (9.4)

^*) ^Diagnostic parameters reported in # per 96 patients. Numbers in parentheses are percentages per 96 Patients

Laboratory parameters in the reported patients with GLB were rather nonspecific (Table 3) and included analytes reflecting blood loss, which was in a few cases corroborated by detecting hematest positive stools (5.2% patients) [3,13,23]. Pathologic parameters of dehydration and electrolyte disturbance following prolonged vomiting were recorded in 12.5% of patients [15,16,23,43,44].

With regard to diagnostic imaging, abdominal ultrasound, reported in 12 cases so far, showed highly echogenic intra-bezoaric air trapping [8,16,18,45-47] and proved to be very sensitive in experienced hands (Table 3). Plain radiography was the most commonly reported method (Table 3): however, in 62 patients investigated this way only 42 GLBs (67.7%) were correctly identified. In the remaining 20 cases with inconclusive plain abdominal radiography and in 25 additional patients, air contrast, clear fluid or opaque contrast medium radiography [45-47], or X-ray video-recording led to the detection of a GLB [23,48-50]. In 2 further patients GLBs were first diagnosed by gastroscopy [19,46] (Table 3). Notwithstanding, in 9 patients GLBs were detected during laparotomy [1,3,12,25]. While gastric perforation was found in 7 of these patients, pneumoperitoneum was identified by plain abdominal X-ray in only 4 of them.

GLB may be an underdiagnosed entity. Firstly, abdominal distension and vomiting/regurgitation are often managed by pausing enteral nutrition and giving intravenous fluids which is also the standard treatment for GLB [2,3,30,32,34,42-44]. This usually leads to complete dissolution and impossibility of documenting the original existence of a GLB. A second cause for underdiagnosis is the frequently short livespan of GLBs - even without treatment - as shown by serial radiological studies [23,31,34,44]. A third argument is low diagnostic sensitivity of physicians who have often initially failed to diagnose GLBs actually present as proven subsequently by repeat evaluation of the same X-ray film [1,2]

Thus, the diagnosis of GLB affords a high degree of experience and suspicion. Yet, bearing in mind the relevant diagnostic criteria (Tables 3 and 4) will permit the correct diagnosis and allow a clear differentiation from other entities with an abdominal mass (Table 5).

**Table 4:** Diagnostic imaging criteria for gastric lactobezoar

Method	Diagnostic criteria
Ultrasound	free-floating intra-gastric mass, moves with patient positioning, intra-bezoaric echogenic air trapping
Plain X-ray	intra-gastric rounded mass only visible when surrounded by sufficient air or fluid, calcifications may be visible
Air-/fluid contrast opaque contrast medium	intra-gastric rounded mass large circular filling defect with mottled surface
X-ray video-imaging	mass moves with patient positioning

**Table 5:** Differential diagnosis of gastric lactobezoar

**Gastrointestinal obstruction**	Other types of gastric bezoar Intestinal or colonic bezoar Pyloric stenosis Intestinal stenosis/atresia Paralytic ileus Meconiumileus Volvulus Intussusception
**Tumors**	Tumor of the liver Tumor of the kidney Neuroblastoma GI-lymphoma
**Respiratory**	Infant respiratory distress syndrome Pneumonia Pneumothorax Diaphragmatic hernia
**Cardiocirculatory**	Cardiac malformation Cardiac insufficiency Sepsis Inadequate supply of fluids

## Management

The therapeutic regimen of nil per mouth, with intravenous fluids alone or in combination with gastric lavage led to a successful outcome in over 85% of cases (Table 6). GLB - resolution was even reported in patients in whom enteral nutrition was continued. Nevertheless, from the bulk of information obtained in this study, a decrease in caloric density seems clearly warranted as soon as first signs of overstraining the individual digestive capacity become apparent. In cases with protracted GLB-dissolution gastric lavage with 20 to 100 ml physiologic saline 4 × daily and protein-cleaving enzymes such as N-acetylcysteine at a concentration of 10 mg × kg ^-1 ^× dose ^-1^, was helpful [16]. An important complementary measure was decompression of the stomach by a nasogastric tube. The success of conservative treatment clearly argues against primary invasive measures. However, caution is warranted when the cause of acute abdomen remains elusive for more than 24 hours as was the case in the majority of the 7 patients with GLB in whom gastric perforation finally turned out to have been a stringent indication for surgery. For 2 additional infants without perforation the need for laparotomy may have been viewed differently if the present knowledge of the state of the art in diagnosis and treatment of GLB had been available [23,43]. Thus surgical treatment is only warranted when 1) a pneumoperitoneum is diagnosed, 2) the cause for an acute abdomen remains unidentified, or 3) an already diagnosed GLB cannot be dissolved by conservative management within 72 hours. In this latter case gastroscopic disintegration may be tried before surgery if the patient is in good physical condition [19,10].

**Table 6:** Reported treatment of patients with gastric lactobezoar

Type of treatment	#/96 patients reported^*)^
No treatment reported	14 (14.6)
Nil per mouth alone	3 (3.1)
Intravenous fluidsand "nil per mouth"	29 (30.2)
Gastric decompression (nasogastric tube)	64 (66.7)
Gastric lavage	16 (16.7)
Gastric lavage with N-acetylcysteine	3 (3.1)
Intravenous fluids+ continued enteral feeds	4 (4.2)
Change to different milk/formula with "normal" concentration	9 (9.4)
Change to different milk/formula with reduced concentration	6 (6.3)
Mechanical disintegration with feeding tube	4 (4.2)
Gastroscopy	2 (2.1)
Surgery	9 (9.4)

^*) ^Types of treatment in 96 patients with gastric lactobezoar. In some patients more than one type of treatment was reported. Numbers in parentheses are percentages of patients managed with the indicated modality.

## Prognosis

GLB has an excellent outcome provided diagnosis and treatment occur in due time [2,10,17,51]. It is important to alert pediatricians to the possibility of GLB being a differential diagnostic entity of acute abdomen. An improved index of suspicion leads to timely diagnosis and reduces severe complications and mortality in these vulnerable kids. Because the response of GLB to treatment can be closely monitored by repeat ultrasound scans conservative measures are a safe approach and will in most cases result in complete GLB-resolution within 2-3 days.

## Perspectives for the future

Prospective controlled studies on the great variety of postulated pathogenetic factors (Table 2) are lacking. Particularly important issues for study comprise:

1) Pathogenetic factors causing GLB: for example volumes and concentrations of gastric contents, pH, osmolality, pepsin activity and indicators of gastric emptying should be studied under defined conditions (e.g. time points during feeding cycle, formula composition, body position during and after feeding) to identify relevant markers.

2) Evaluation of risk factors associated with gastric perforation: prematurity was found to be one of them. However, from the existing publications it was impossible to decide if a GLB or a nasogastric tube - or other factors including a delay in diagnosis - were responsible for the perforation. Therefore prospective evaluation of these parameters is clearly warranted.

## Abreviations

BUN: blood urea nitrogen; DRI: dietary reference intake; GI: gastrointestinal; GLB: gastric lactobezoar; LBW: low birth weight; LCT: long chain triglyceride; MCT: medium chain triglyceride

## Competing interests

The authors declare that they have no competing interests.

## Authors' contributions

PH-E drafted the manuscript. IG contributed information on diagnostic imaging. AK-F described mechanisms causing anemia in GLB patients. RT elaborated on neonatal pathophysiology. CN collected and analysed data from the reviewed literature. TM provided insights in pathophysiological mechanisms of lactobezoar formation. BM revised the manuscript. VJ and SSB compiled information on nutritional aspects supporting the formation of GLB. All authors read and approved the final manuscript.
